# Global incidence, prevalence and disease burden of silicosis: 30 years’ overview and forecasted trends

**DOI:** 10.1186/s12889-023-16295-2

**Published:** 2023-07-17

**Authors:** Xin Liu, Qingtao Jiang, Peihong Wu, Lei Han, Peng Zhou

**Affiliations:** 1 Institute of Occupational Disease Prevention, Jiangsu Provincial Center for Disease Control and Prevention, Nanjing, China; 2 Jiangsu Province Engineering Research Center of Health Emergency, Nanjing, China; 3 Department of clinical medicine, Jiangsu Health Vocational College, Nanjing, China

**Keywords:** Global burden of Disease (GBD), Prediction, Silicosis

## Abstract

**Background:**

Globally, silicosis accounts for 90% of all pneumoconiosis cases and is a serious public health issue. It is characterized by progressive inflammation and irreversible pulmonary fibrosis. A comprehensive analysis at temporal, spatial and population levels with the most updated data from GBD 2019 is provided in this study to estimate the disease burden of silicosis from 1990 to 2019 and make predictions to 2029.

**Methods:**

We delineated silicosis data on incidence, prevalence, and disability-adjusted life years (DALYs) as well as age-standardized rates (ASRs) across 30 years from GBD 2019. Joinpoint regression analysis was employed to detect temporal changes and estimate annual percentage change (APC) of each trend segment. Measures were stratified by time, location, age, and sociodemographic index (SDI). Back propagation artificial neural network (BP-ANN) model was applied to elaborate ASR trends from 1990 to 2019 and projections to the next 10 years.

**Results:**

Globally, silicosis incident, prevalent cases, and DALYs increased by 64.6%, 91.4%, and 20.8%, respectively. However, all the corresponding ASRs showed overall downward trends with an estimated average APC (AAPC) of -0.5(-0.7 to -0.3), -0.2(-0.5 to 0.0), and − 2.0(-2.2 to -1.8), respectively. Middle and high-middle SDI regions carried the heaviest disease burden. The highest disease burden of silicosis was mainly transferred to the older from 1990 to 2019. The trend of ASRs demonstrated a rapid decline between 2005 and 2019, followed by a continuous decline until 2029.

**Conclusion:**

Though disease burden of silicosis has been on a decline in general from 1990 to 2019, which shows a promising prospect but cannot be ignored. We should pay more attention to implementing preventive tactics and improving the life quality of present sufferers.

## Background

Silicosis is a typical pneumoconiosis and common in workers who have large chances to be exposed to free crystalline silica (SiO_2_) during long-term occupational activities [1]. Generally, silicosis is characterized by the development of irreversible and progressive pulmonary fibrosis [2]. The pulmonary function injury and distinctive respiratory symptoms of silicosis include chest tightness, shortness of breath, chest pain, cough, hemoptysis, sputum expectoration and subsequent complications such as respiratory tract infection, pneumothorax, and tuberculosis may arise [3–5].

Despite the strict supervision and management of SiO_2_ industry, the number of workers occupationally exposed to silica is up to 1.7 million in USA and much more around the world every year [6]. Much efforts has been made in recent years, besides lung transplantation offered for a few patients, there is still no effective countermeasures to date [7].

Burden of disease (BOD) is a series of epidemiological indicators to evaluate the impact of disease on social healthcare. Some previous studies focused on the death trends owing to silicosis, revealing a potentially serious burden worldwide [5, 8]. However, as a typical chronic disease, limited attentions has been paid to assessing data of great importance for expanding our specific understanding of silicosis incidence and prevalence. Updating estimates and deconstructing the burden of silicosis by age, time, and location is of paramount importance but has been overlooked. Moreover, a long-term prediction for BOD of silicosis remains scarce in the literature.

The Global Burden of Diseases Study (GBD) 2019 covered 369 types of diseases and injuries across 204 countries and territories, which can help promote the decoding of landscape and dynamics underlying silicosis from a unique perspective [9]. Therefore, we attempted to collect in-depth information on the BOD indicators of silicosis from GBD 2019 in this study. Furtherly, we aim to present results for ASRs and corresponding cases or numbers, analyze AAPC and utilize machine learning tools to make predictions of silicosis based on the spatial, temporal and population distributions, in order to evaluate the epidemic trends of silicosis.

## Materials and methods

### Study data

GBD 2019, covering 369 diseases and injuries in 204 countries and territories from 1990 to 2019, provides comparable and systematic estimations of data by the Institute of Health Metrics and Evaluation (IHME) [10, 11]. The estimates of silicosis were predominantly derived from systematic reviews, inpatient hospital reports and reported claims-based data, and then analyzed with demographic methods to complete missing values [12–14]. A Bayesian meta-regression model (DisMod-MRV.2.1), as the main method of estimation in the GBD, is a type of mixed-effect model that borrows information across age, time and locations. This model not only controls and adjusts biases in the data but also integrates multiple data sources to generate comprehensive and unified estimates of levels and trends [15].

The 204 countries and territories were divided into 5 categories according to SDI which covers lag-distribution income per capita, education, and total fertility rates [16, 17]. The range of SDI was 0 to 1. We collected cases or numbers and corresponding ASRs on incidence, prevalence and DALYs for silicosis, based on online available public data from the Global Health Data Exchange website (http://ghdx.healthdata.org/gbd-results-tool), according to GBD’s operation instruction.

### Statistical analysis

#### Data management

The ASR is reported per 100,000 people or person-years with 95% uncertainty intervals (UIs). We used the Joinpoint regression software (V.4.7.0.0) to calculate measures of ‘AAPC of ASR’, which is a summary of ASR trends over a prespecified interval. Traditional regression models primarily fit and evaluate the overall trend of disease distribution within a given time range from a global perspective, unable to capture local variations, while Joinpoint model is to establish segmented regression based on the temporal characteristics of disease distribution. By using several joinpoints, the study period is divided into different intervals, and trend fitting as well as optimization are performed on each interval. This enables a more detailed assessment of the specific disease change characteristics within different intervals of the overall time range. These results are presented as AAPC accompanied by corresponding 95% CI. Briefly, if the AAPC and its 95% CI were both > 0, the ASR was indicated to have had a corresponding increasing trend. In contrast, if they were both < 0, the ASR was deemed to have had a corresponding decreasing trend. In our study, silicosis incidence, prevalence and DALY were analyzed by applying the population-attributable fraction for age, location and year.

#### Prediction model

BP-ANN model, a multilayer feedforward network trained by error back propagation algorithm [18], is innovatively applied to describe time course of ASR and predict further trends. The BP-ANN model architecture includes the input layer, the hidden layer, and the output layer. Each layer has at least one neuron, which connects to neurons in different layers. This structure is characterized by simplicity and clarity, allowing each neuron to establish an appropriate linear or non-linear relationship between input and output [19, 20]. According to the training mode of BP-ANN in current study, the input layer and hidden layer have 10 nodes respectively. The number of nodes in the output layer is 1. The activation function of hidden layer as well as output layer is tansig and logsig. The learning rate was set as 0.05, while the convergence error limit is 0.005.

All data analysis and visualization were produced by R (version 4.0.3) based on the dataset generated by GBD 2019.

## Results

### Global and regional burden and trends

**Fig. 1:**
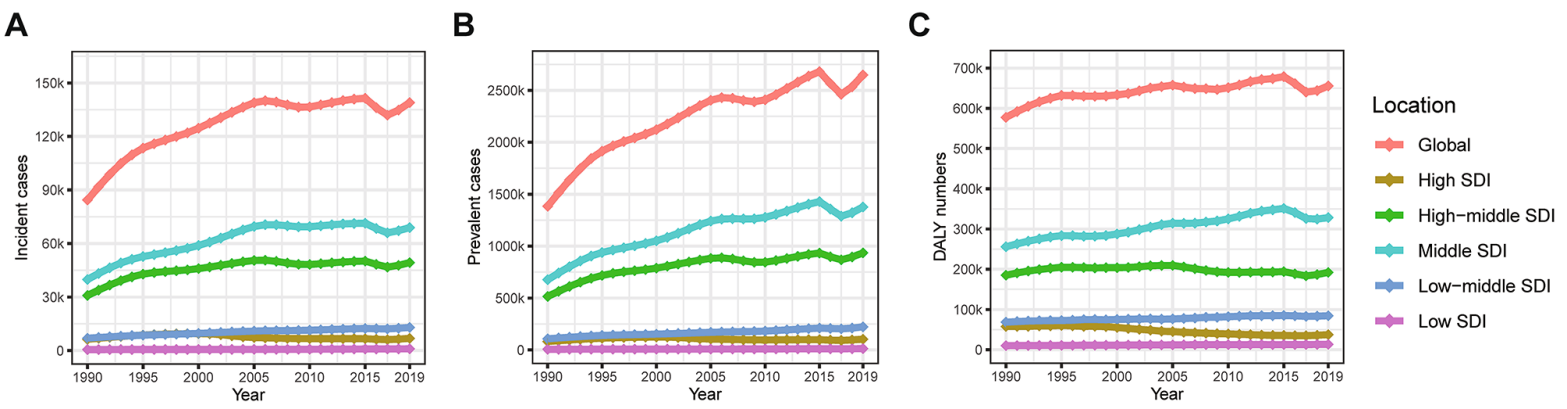
The changes in the incident, prevalent cases and DALY numbers of silicosis from 1990 to 2019. (A) Incident cases; (B) Prevalent cases; (C) DALY numbers

**Fig. 2:**
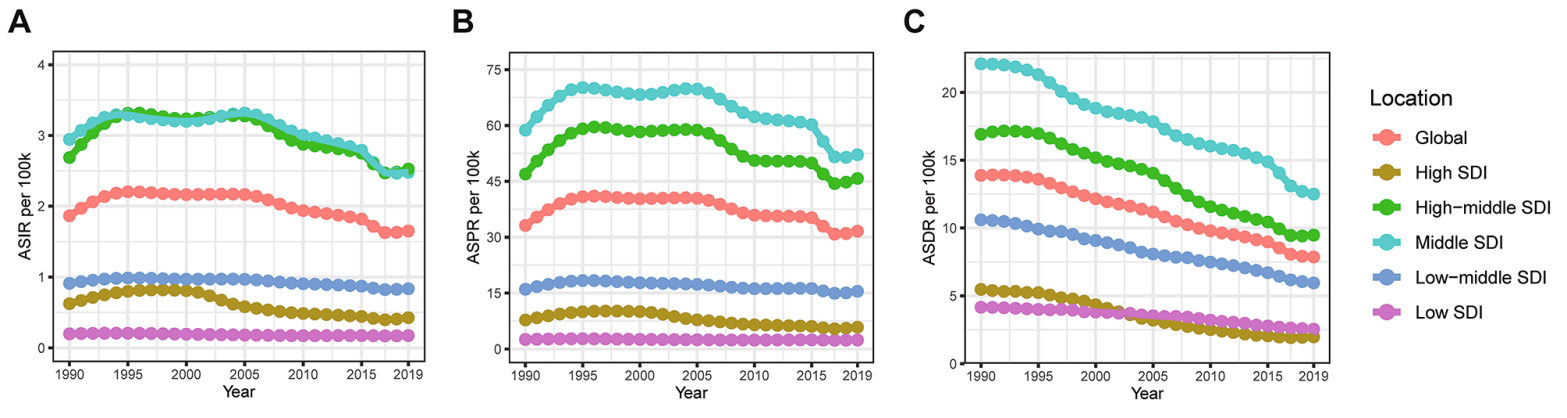
The ASRs of silicosis from 1990 to 2019. **(A)** ASIR; **(B)** ASPR; **(C)** ASDR.

**Fig. 3:**
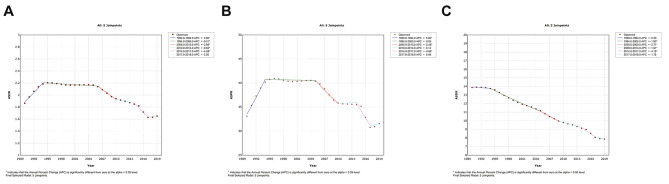
Trend of ASRs by Joinpoint Regression (5 joinpoints), 1990–2019. **(A)** ASIR; **(B)** ASPR; **(C)** ASDR.

In the global analysis of 204 countries and different regions, we assessed the trends in numbers and ASRs of silicosis between 1990 and 2019. Globally, the incident cases of silicosis increased by a measure of 64.6%, from 84,821 cases in 1990 to 138,965 cases in 2019 (Table 1; Fig. 1A). The age-standardized incidence rate (ASIR) was found to have decreased by an average of 0.5% per year (*p* < 0.05) in the same period (from 1.86/100,000 in 1990 to 1.65/100,000 in 2019; Table 1; Fig. 2A). As shown in Table 2; Fig. 1B, prevalent cases of silicosis grew from 1,383,913 in 1990 to 2,648,973 in 2019, which showed an increase of 91.4%. Whereas the age-standardized prevalence rate (ASPR) declined from 33.13/100,000 in 1990 to 31.60/100,000 in 2019 by an average of 0.2% each year (Table 2; Fig. 2B). Meanwhile, DALYs due to silicosis rose by 20.8% from 577,390 to 1990 to 655,763 in 2019 (Table 3; Fig. 1C). The age-standardized DALY rate (ASDR) was reduced from 13.88/100,000 in 1990 to 7.87/100,000 in 2019 with an AAPC of 2.0% ((*p* < 0.05, Table 3; Fig. 2C). As shown in Fig. 3, the ASIR and ASPR of silicosis ascended with different APCs between 1990 and 1995 (APC = 3.98% and 5.00%, *p* < 0.05), reaching a 10-year steady period followed with a continuous declination. For overall ASDR, the constant decreasing trend across the study period was observed. The highest silicosis burden occurred in regions with middle SDI and high-middle SDI, followed by low-middle SDI and high SDI, while the lowest was found in low SDI (Figs. 1 and 2).

**Table 1:** Global trends on incidence of silicosis from 1990 to 2019

Location	ASIR, 1990 (×1/10^5^)	ASIR, 2019 (×1/10^5^)	Percentage changes, 1990–2019 (%)	AAPCs, 1990–2019 (%)	Incident cases, 1990	Incident cases, 2019
Global	1.86(1.51–2.29)	1.65(1.36–1.98)	-11.29	-0.5*(-0.7~-0.3)	84,421	138,965
High SDI	0.62(0.48–0.81)	0.42(0.36–0.50)	-32.26	-1.5*(-1.9~-1.1)	6320	6841
High-middle SDI	2.69(2.19–3.32)	2.53(2.08–3.02)	-5.95	-0.3*(-0.6~-0.1)	30,885	49,260
Middle SDI	2.94(2.36–3.66)	2.48(2.01–2.96)	-15.65	-0.7*(-1.0~-0.4)	39,793	68,908
Low-middle SDI	0.91(0.75–1.08)	0.83(0.69-1.00)	-8.79	-0.4*(-0.5~-0.2)	6903	12,986
Low SDI	0.20(0.16–0.25)	0.17(0.14–0.22)	-15.00	-0.5*(-0.6~-0.4)	520	970

Note: ASIR: Age-standardized incidence rate; AAPCs: Average Annual Percentage Change; SDI: Socio-Demographic Index. The data in parentheses are 95% uncertainty intervals. *P < 0.05

**Table 2:** Global trends on prevalence of silicosis from 1990 to 2019

Location	ASPR, 1990 (×1/10^5^)	ASPR, 2019 (×1/10^5^)	Percentage changes, 1990–2019 (%)	AAPCs, 1990–2019 (%)	Prevalent cases, 1990	Prevalent cases, 2019
Global	33.13(26.71–42.19)	31.60(26.06–37.87)	-4.62	-0.2(-0.5 ~ 0.0)	1,383,913	2,648,973
High SDI	7.76(5.98–10.46)	5.82(4.93–6.93)	-24.91	-1.0*(-1.6~-0.5)	80,954	103,106
High-middle SDI	46.94(37.74–58.93)	45.75(37.80-55.27)	-2.52	-0.1(-0.4 ~ 0.2)	514,816	934,452
Middle SDI	58.75(46.76–75.94)	52.14(42.61–63.10)	-11.25	-0.4*(-0.9~-0.0)	675,240	1,376,876
Low-middle SDI	16.02(13.15–19.17)	15.47(12.89–18.35)	-3.45	-0.2*(-0.3~-0.0)	106,822	222,347
Low SDI	2.55(2.06–3.14)	2.40(1.93–2.99)	-5.78	-0.2*(-0.4~-0.1)	6016	12,114

Note: ASPR: Age-standardized prevalence rate; AAPCs: Average Annual Percentage Change; SDI: Socio-Demographic Index. The data in parentheses are 95% uncertainty intervals. *P < 0.05

**Table 3:** Global trends on DALY of silicosis from 1990 to 2019

Location	ASDR, 1990 (×1/10^5^)	ASDR, 2019 (×1/10^5^)	Percentage changes, 1990–2019 (%)	AAPCs, 1990–2019 (%)	DALY, 1990	DALY, 2019
Global	13.88(10.81-17.00)	7.87(6.25–9.96)	-43.30	-2.0*(-2.2~-1.8)	577,390	655,763
High SDI	5.48(4.88–6.33)	1.98(1.61–2.82)	-63.87	-3.5*(-3.8~-3.2)	57,818	37,443
High-middle SDI	16.91(13.65–20.37)	9.48(7.13–12.40)	-43.94	-2.1*(-2.3~-1.8)	185,018	192,032
Middle SDI	22.11(16.01–28.27)	12.50(9.70-16.09)	-43.46	-2.0*(-2.3~-1.7)	255,802	328,415
Low-middle SDI	10.59(5.91–14.19)	5.95(4.47–7.49)	-43.81	-2.0*(-2.2~-1.9)	68,609	84,548
Low SDI	4.16(0.99–7.71)	2.54(0.96–3.98)	-38.94	-1.7*(-1.9~-1.5)	10,079	13,283

Note: ASDR: Age-standardized DALY rate; AAPCs: Average Annual Percentage Change; SDI: Socio-Demographic Index. The data in parentheses are 95% uncertainty intervals. *P < 0.05

### Age‑specific burden in 1990 and 2019

**Fig. 4:**
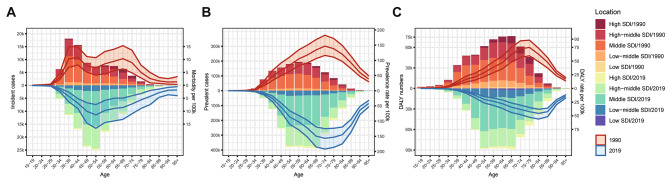
The age distributions on incidence, prevalence and DALY of silicosis by SDI regions in 1990 and 2019. **(A)** Incidence of silicosis; **(B)** Prevalence of silicosis; **(C)** DALY of silicosis

As revealed in Fig. 4A, the age-specific incident cases of silicosis increased rapidly after the age of 25, reaching the zenith in the age group 35–39 at 1990 and 50–54 at 2019, whereas the age-specific morbidity peaked in 35–39 and 65–69 age groups at 1990 and 50–54 at 2019 respectively. The age-specific prevalent cases of silicosis rose sharply after the age of 30, reaching the highest point in the age group 55–64. Meanwhile, the peak prevalence rate of silicosis was observed in the age group 70–74 (Fig. 4B). Moreover, the DALY numbers due to silicosis increased monotonically at a steady pace among people aged < 69 years and then decreased, while the DALY rate reached its peak at the age of 75–79 in 1990, which was an age interval ahead of 2019 (Fig. 4C). As for burden of silicosis attributable to SDI regions, middle SDI and high-middle SDI accounted for the majority of all age groups.

### Country‑specific burden in 2019

**Fig. 5:**
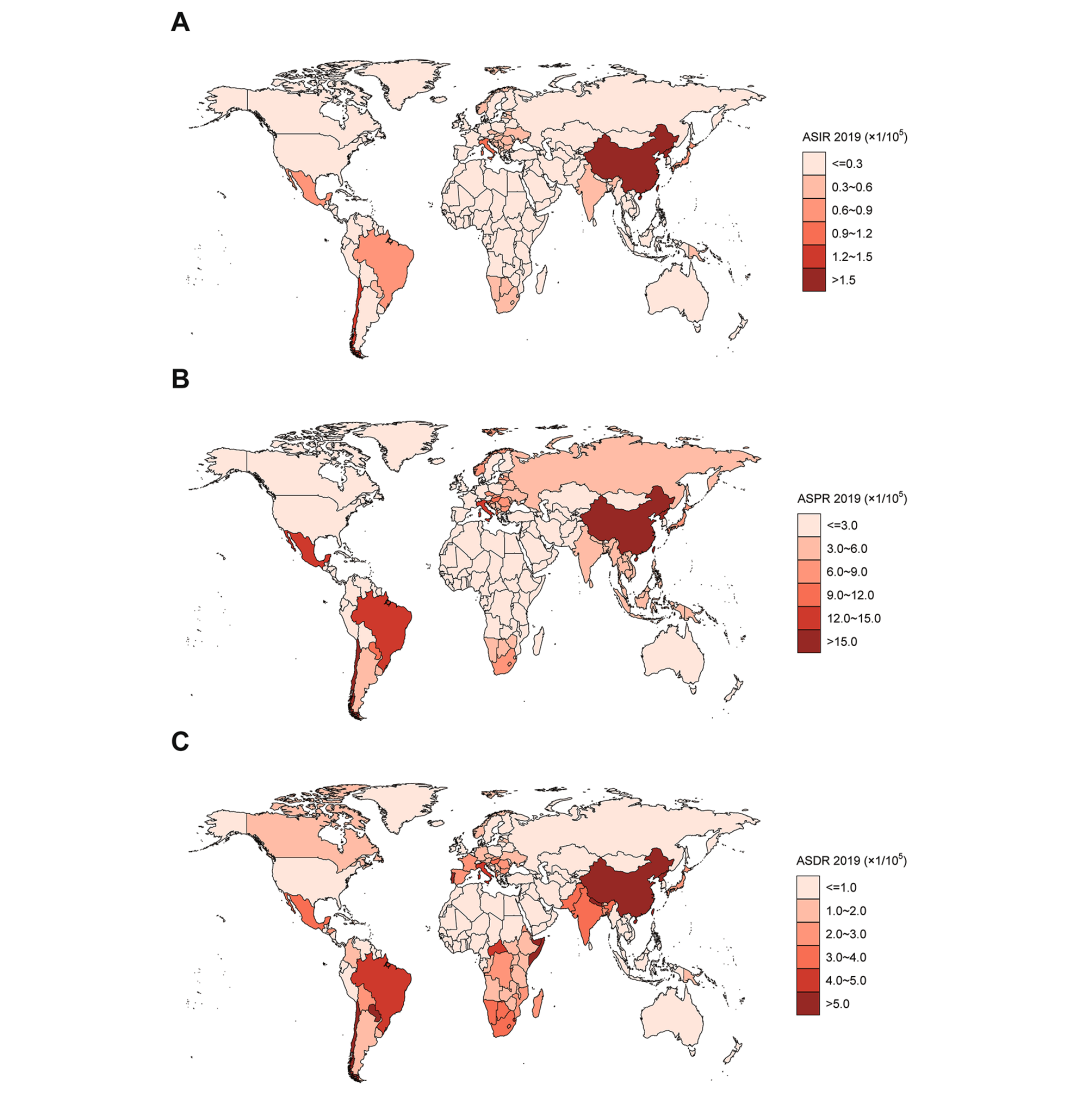
The spatial distribution on ASRs of silicosis in 2019. **(A)** ASIR; **(B)** ASPR; **(C)** ASDR.

From a global point of view, the ASRs attributable to silicosis in 2019 was quite different among countries. The ASIR varied from < 0.001/100,000 in Iceland to 5.92/100,000 in China (Fig. 5A), of which 4 countries (Italy, Chile, North Korea and China) were over 1/100,000 and 101 countries were below 0.1/100,000. Similarly, Iceland was also the lowest with regard to ASPR, while China was the highest (113.15/100,000) followed by North Korea, Chile, Mexico, Italy, Brazil, Palau, Albania and Slovenia where rates were estimated to exceed 10/100,000 (Fig. 5B). As for ASDR, the lowest rate of silicosis was still observed in Iceland whereas China showed the highest rate, followed by North Korea, Palau and Chile (Fig. 5C). Overall, Iceland and China took the lowest and highest places of ASRs for silicosis respectively across the world in 2019.

### Predictions for silicosis in next 10 years

**Fig. 6:**
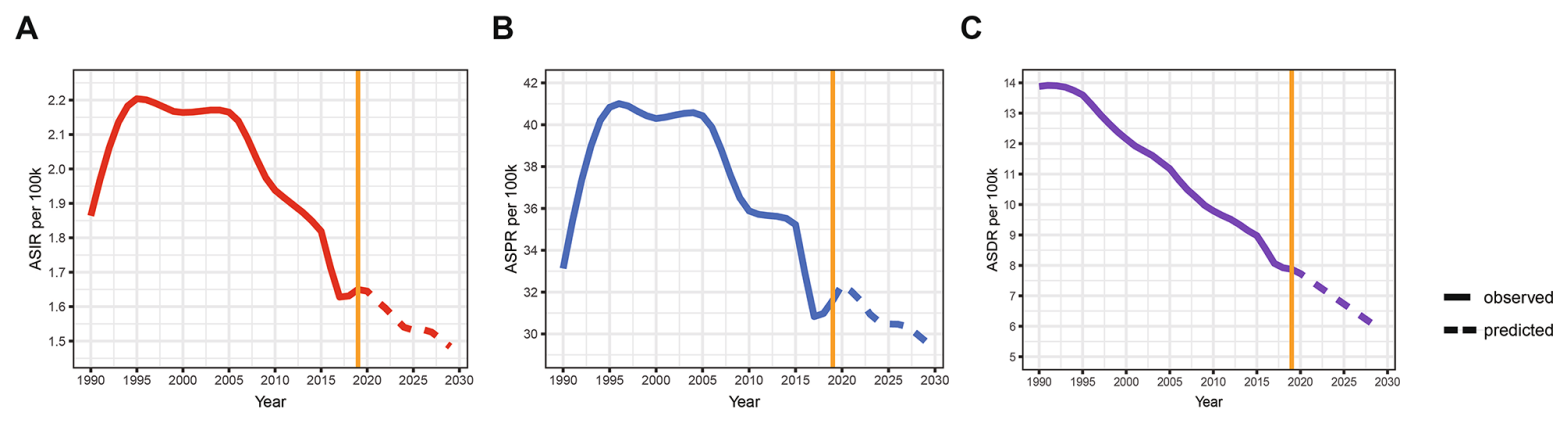
Trends in observed and predicted ASRs of silicosis from 1990 to 2029. **(A)** ASIR; **(B)** ASPR; **(C)** ASDR.

BP-ANN model was established via R package ‘AMORE’. Figure 5 shows the observed and predicted trends for ASIR, ASPR and ASDR of silicosis from 1990 to 2029 globally. We unveiled a substantial decline in ASIR after the year of 2005, and this rapid decline was predicted to continue and reach approximately 1.48/100,000 in 2029 (Fig. 6A). The ASPR of silicosis also descended in the prediction period except for a slight fluctuation during 2018–2020 (Fig. 6B). Similarly, an obvious descent curve of ASDR was observed after the year of 2019, reaching nearly 5.93/100,000 until 2029 (Fig. 6 C). Our predictive results indicate that all components of the silicosis BOD will decrease continuously in the next 10-year cycle.

## Discussion

This study adopted the methods and data of GBD 2019 to survey and evaluate the BOD of silicosis in the global population, forming a unified and comparable result with BOD of other diseases and other types of pneumoconiosis globally. In accordance with the International Statistical Classification of Diseases and Related Health Problems (ICD-10), the specific diseases included in this analysis mainly include pneumoconiosis due to dust containing silica (J62), pneumoconiosis due to talc dust (J62.0) and pneumoconiosis due to other dust containing silica (J62.8) [21].

Silicosis is the leading cause of BOD from pneumoconiosis [22, 23]. From 1990 to 2019, the incident and prevalent cases of silicosis increased by 64.6% and 91.4% respectively, while DALY numbers only rose by 20.8%. In the meantime, ASIR and ASPR have decreased by 0.5% and 0.2% on average every year, while ASDR has declined by more than 2.0% yearly, which indicated that the world has made positive achievements in the prevention and treatment of silicosis. As we know, the prevalence status is more important for assessing the disease burden of chronic diseases. For the first time, we estimated the prevalent cases and rates of silicosis in worldwide over the past 30 years, revealing the current situation of high prevalence of silicosis. It could be inferred that the reduction of BOD should begin by improving the quality of life (QOL) among affected individuals.

In terms of age distribution, the peak of incidence rate in the past 30 years had shifted from 35 to 39 to 50–54, which implied that the prevention and control measures delay the occurrence of silicosis. The highest prevalence rate was observed in the 70–74 age group, while the peak DALY rate occurred in the 75–84 age group. In addition, the incident, prevalent cases and DALY numbers all reached zenith after the age of 50 in 2019, which may be associated with the aging of global population. As shown in Fig. 4, the BOD of silicosis was mainly borne by countries with middle SDI and high-middle SDI. The industrial systems of these countries are relatively well developed and some workplaces with occupational hazards of crystalline silica dust were involved. China, Brazil and South Africa, for example, are middle SDI countries where the main source of silicosis is the mining industry [24–26]. While in Spain, as well as other high-middle SDI European countries, occupational exposure to secondary processing of quartz or new artificial stone has led to silicosis among workers [27, 28]. These countries still face a heavy BOD.

We noticed that silicosis had substantial declines in ASIR and ASPR after 2005 despite of slight fluctuations, and these rapid declines were predicted to continue in the following 10 years. Meanwhile, in our prediction model, a steady downward trend of ASDR was identified constantly until 2029. Though the prevalence of silicosis remains high at present, the improvement of regimens has, to some extent, attenuated the patients’ outcome by reducing the years of life lost, a key component of DALY. Therefore, the above factors may contribute to a stable decline of ASDR in a long period.

China has the heaviest BOD of silicosis and only 15.9 thousand new cases of pneumoconiosis was reported by the government in 2019 [8]. The underestimation may due to the following reasons. First, the cases occurring in a large number of small or disorganized enterprises are not reported through the formal way. Second, the notifiable occupational diseases reported by government require adherence to stringent diagnostic procedures. Furthermore, the mobility of migrant workers, coupled with the long incubation period of silicosis, poses challenges to to the case report adhesion. There are approximately 280 million migrant workers in China, who may be employed in mines, construction and manufacturing industries [29]. Their mobility results in loose occupational health supervision.

Generally, silicosis BOD is on a downward trend but cannot be ignored. Middle SDI and high-middle SDI countries suffer much more than other SDI regions. The health departments and policy makers should pay more attention to implementing preventive tactics and improving the QOL of present sufferers. The employers have to persist in strengthening the control of workplace dust exposure, while workers should wear personal protective equipment properly. Regular medical examinations as well as health education and training should be carried out effectively.

### Limitation

Several limitations should be mentioned before further interpreting the results. First, all the data used in our study are from GBD 2019, which were gathered from different sources [30]. Thus, inconsistencies and incompatibilities between these data might exist, which may cause bias. Second, this study was unable to estimate the situation of people with silicosis that was misdiagnosed by other pulmonary complications, hence the true burden of silicosis may be underestimated. Finally, these data were relied on calculations of surveillance data, and these indicators may have a lag in the estimation of current BOD.

## Conclusion

Silicosis continues to be one of the most important occupational health issues and causes a potentially serious BOD worldwide. Though ASRs have experienced some fluctuations from 1990 to 2019, the trends may still be expected to descend steadily in the future. Greater efforts should be devoted to the development of effective drugs or treatments, as well as improving the QOL for individuals already affected by silicosis.

## Data Availability

The datasets generated and/or analyzed during the current study are available in the GBD Data Tool repository (http://ghdx.healthdata.org/gbd-results-tool). This public link to GBD database is open, and the use of data does not require additional consent from IHME.
